# Effects of physical exercise on macular vessel density and choroidal thickness in children

**DOI:** 10.1038/s41598-021-81770-y

**Published:** 2021-01-21

**Authors:** Shufeng Li, Yiguo Pan, Jingjing Xu, Xue Li, Daniel P. Spiegel, Jinhua Bao, Hao Chen

**Affiliations:** 1 Eye Hospital, School of Ophthalmology and Optometry, Wenzhou Medical University, 270 Xueyuan Road, Wenzhou, 325027 Zhejiang China; 2 R&D Vision Sciences AMERA, Essilor International, Singapore, Singapore

**Keywords:** Physiology, Medical research

## Abstract

We used swept-source (SS) optical coherence tomography (OCT) and OCT angiography (OCTA) to investigate the effects of moderate physical exercise on retinal and choroidal vessel densities (VDs) and thicknesses in children. One eye in each of 40 myopic children (mean age, 11.70 years) and 18 emmetropic children (mean age, 11.06 years) were included. SS-OCT 6 × 6-mm radial scans and SS-OCTA 3 × 3-mm images were centered on the macula. Heart rate (HR), systolic and diastolic blood pressure, and intraocular pressure (IOP) were recorded before and immediately after a 20-min stationary cycling exercise and after a 30-min rest. The subfoveal choroidal thickness (SFCT), choroidal thickness (CT), and VD at the superficial and deep retinal layers, choriocapillaris, and deeper choroidal vessels were determined. SFCT and CT were significantly lower at all locations immediately after exercise (*p* < 0.001) and did not fully recover after rest (*p* < 0.05). VD was lower in the deep retinal layer after exercise (*p* = 0.02) and higher in the superficial layer after rest (*p* = 0.03) in myopic eyes while it was higher in the superficial (*p* < 0.01) and deep layer (*p* < 0.01) after rest in emmetropic eyes. No significant exercise-related changes in the superficial retinal VD, choroidal VD, or IOP were observed. ΔCT% and ΔSFCT% were significantly correlated with increases in HR in myopic group (*p* = 0.04 and *p* = 0.03, respectively). Exercise increased retinal VD after rest in emmetropic eyes, and caused significant CT thinning that lasted for at least 30 min in both emmetropic and myopic eyes.

## Introduction

Physical exercise has positive effects in children^1,2^. The benefits of exercise can be attributed to several mechanisms, including increased cardiorespiratory fitness and the strategic redistribution of blood flow that occurs through vasodilatation of the heart and skeletal muscles and/or vasoconstriction of the skin and splanchnic tissues^3^. Proteins, peptides, enzymes, and metabolites released from one organ can affect the metabolism of other organs as the blood flow becomes redistributed during exercise^4^. While the effects of physical exercise have been studied in many organs of both adults and children, how it affects the eyes in children is relatively unknown. The retina and choroid are densely vascularized, and appropriate levels of ocular blood flow are indispensable to normal visual performance^5^. Given that exercise changes the cardiovascular system throughout the body, changes in blood pressure (BP) and heart rate (HR) are likely to affect the retina and choroid.

Some previous studies have investigated the associations between exercise-induced alterations in the systemic circulation and changes in the retina and choroid in adults, but the results have been controversial^6–10^. Alnawaiseh et al.^10^ reported that peripapillary and parafoveal flow densities decreased significantly after exercise. Vo Kim et al.^6^ also reported that the vessel density (VD) at the level of the retinal superficial layer, from 3 μm below the internal limiting membrane (ILM) to 15 μm below the inner plexiform layer (IPL), were decreased after exercise while the fractal dimension in the retinal deep layer, from 15 to 70 μm below the IPL, were significantly increased. Hayashi et al.^7^ found that the blood flow and vascular conductance of retinal arterioles remained stable while ocular blood flow increased in the retina and choroid during physical exercise. Changes reported for choroidal thickness (CT) associated with exercise were inconsistent among previous studies. CT remained stable in most of these reports^11,12^ but increased in others^13^.

Although most epidemiological studies have demonstrated that the benefits for children of outdoor activity are derived from exposure to light, the effects of exercise on eye structure and function have not been well studied^14^. The aim of our study was to evaluate the impact of exercise-induced changes in retinal VD (RVD), choroidal VD (CVD), and CT in children. We used swept-source optical coherence tomography angiography (SS-OCTA) and swept-source optical coherence tomography (SS-OCT) to measure the effects of exercise on RVD, CVD, and CT, and we evaluated correlations between these ocular parameters and systemic physiological parameters.

## Results

Fifty-eight eyes from 58 children were enrolled in this study (ages 9–13 years): 40 myopic eyes (mean spherical equivalent (SE): − 3.27 ± 1.16 diopter (D)), 18 emmetropic eyes (mean SE: 0.03 ± 0.31 D). All of the subjects were free of any ocular or systemic disease and were not taking any medication.

### Changes in systemic hemodynamic parameters

**Figure 1:**
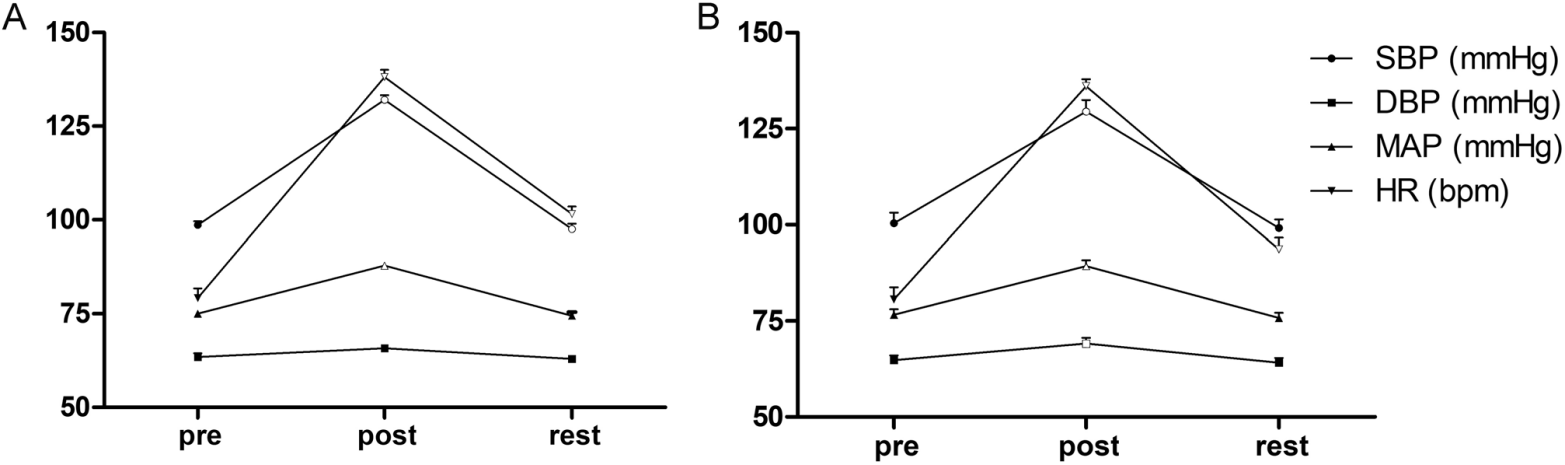
Changes in systemic dynamic parameters among measurements obtained before the 20 min of cycling exercise, immediately after the exercise, and after the 30-min rest period. (**A**) Systemic dynamic parameters of myopic group. (**B**) Systemic dynamic parameters of emmetropic group. The error bars represent the standard error of the mean, all compared with the data before exercise (hollow symbols represent p < 0.001 vs. pre-exercise). *SBP* systolic blood pressure; *DBP* diastolic blood pressure, *MAP* mean arterial pressure, *HR* heart rate.

In myopic group, the mean systolic blood pressure (SBP), mean arterial pressure (MAP), and HR were significantly higher after 20 min of exercise than before, changing from 98.65 ± 8.16 to 132.0 ± 9.77 mmHg, from 75.03 ± 5.70 to 87.85 ± 6.00 mmHg, and from 79.23 ± 12.45 to 138.15 ± 11.19 bpm, respectively (all *p* < 0.001, Fig. 1A). After a 30-min rest, these values returned close to the pre-exercise levels, except for HR, 101.58 ± 10.15 bpm, which was significantly decreased but still higher than at baseline (*p* < 0.001). In addition, HR increased to more than 66% of the age-dependent maximal HR (estimated as 220 − age^15^). The IOP after exercise, 17.25 ± 2.75 mmHg, and after the rest period, 17.83 ± 2.73 mmHg, were not significantly different from the pre-exercise value, 17.23 ± 2.03 mmHg (*p* = 0.947and *p* = 0.106, respectively). The control group completed the same exercise protocol with similar changes (Fig. 1B).

### Changes in SS-OCT and SS-OCTA parameters

**Figure 2:**
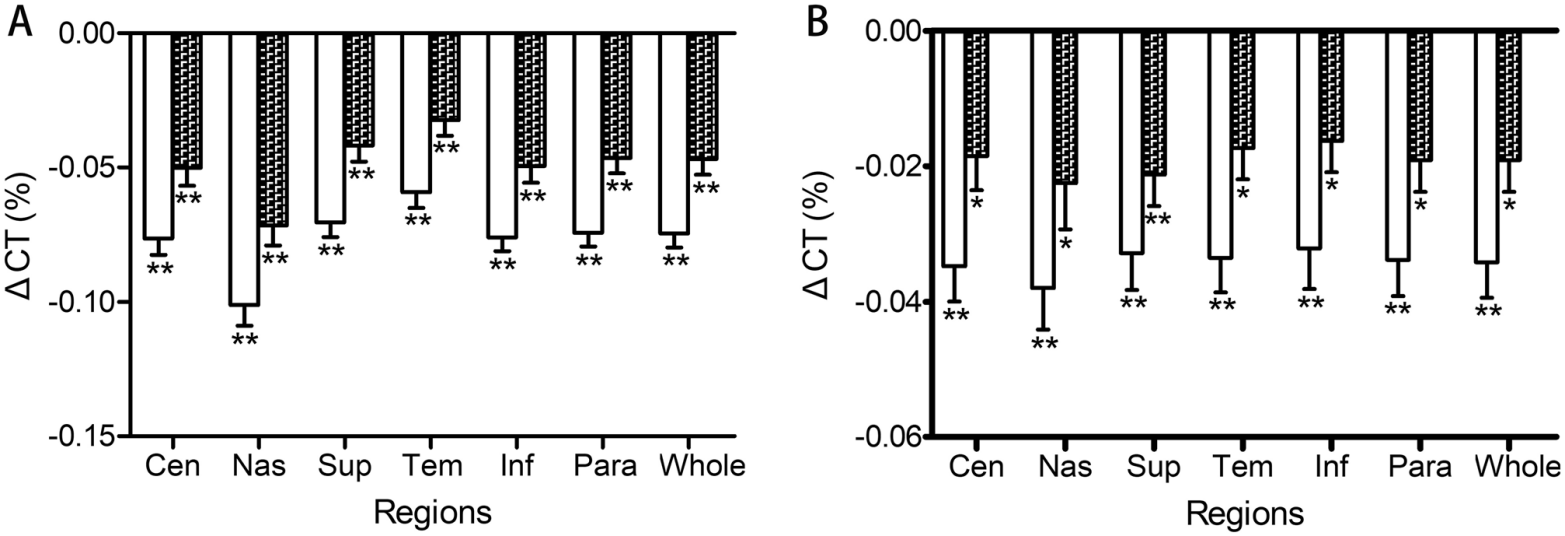
Changes in CT% at different locations immediately after exercise and after rest. (**A**) Changes of CT% at different locations of myopic eyes. (**B**) Changes of CT% at different locations of emmetropic eyes. The error bars represent the standard error of the mean (*, *p* < 0.05; **, *p* < 0.001 vs. pre-exercise, respectively). *CT* choroidal thickness; *Cen* central region; *Nas* nasal; *Sup* superior; *Tem* temporal; *Inf* inferior represents the mean data of inner and outer regions. Para, parafoveal region; Whole, whole region.

The overall CT was significantly reduced after exercise in both myopic and emmetropic eyes, changing from 227.45 ± 41.76 to 210.53 ± 39.46 µm in myopic eyes and from 271.14 ± 54.89 µm to 261.86 ± 53.37 in emmetropic eyes (both *p* < 0.001). After rest, the thickness partially recovered but still remained thinner than before exercise, 218.40 ± 40.08 µm in myopic eyes (*p* < 0.01) and 265.65 ± 52.31 µm in emmetropic eyes (*p* < 0.05, Fig. 2). Similarly, subfoveal choroidal thickness (SFCT) was significantly lower after exercise than before, changing from 226.60 ± 47.08 to 212.33 ± 45.22 µm in myopic eyes (*p* < 0.001), from 277.41 ± 58.75 to 265.43 ± 54.86 µm in emmetropic eyes. The SFCT partially recovered after rest but was still thinner than that before exercise, 217.70 ± 45.18 µm in myopic eyes and 271.54 ± 57.03 µm in emmetropic eyes (*p* < 0.05). Significantly, the changes in CT% of myopic eyes were larger than that of emmetropic eyes (− 7.45 ± 3.32% vs. − 3.77 ± 1.58%, *p* = 0.007).

In the superficial layer of the retina in myopic eyes, the pre-exercise overall and parafoveal VD values, 43.35 ± 1.63% and 48.88 ± 1.57% (Table 1), did not change significantly after the exercise period (*p* = 0.46 and *p* = 0.67 respectively). However, after the 30-min rest period, the parafoveal VD, 49.20 ± 1.50%, were higher than the pre-exercise values (*p* = 0.03). There were no significant changes in the superficial foveal VD during the study. In the deep layer of the retina in myopic eyes, the overall VD and parafoveal VD values was significantly lower after exercise than before exercise, changing from 44.20 ± 1.41% and 50.90 ± 1.71% to 43.87 ± 1.55% and 50.54 ± 1.75% (*p* = 0.02 and *p* = 0.03 respectively), and they returned approximately to the pre-exercise level after rest (Table 1). The foveal VDs of the deep layer of the retina in myopic eyes remained stable during the study. In emmetropic eyes, after the 30-min rest, the overall and parafoveal VD values in both the superficial and deep layers were higher than the pre-exercise values (*p* < 0.01 and *p* = 0.02 in superficial layer, both *p* < 0.01 in the deep layer, respectively).

**Table 1:** Changes in RVD in the superficial and deep layers of myopic and emmetropic eyes before exercise, after 20 min of exercise, and after 30 min of rest.

	Variables	Pre-exercise (95% CI)	Post-exercise (95% CI)	Rest (95% CI)	*P* Value (post–pre/rest-pre)
Myopia (N = 40)	Superficial overall RVD(%)	43.35 ± 1.63 (42.83–43.87)	43.25 ± 1.50 (42.77–43.73)	43.63 ± 1.45 (43.17–44.09)	0.462/0.061
	Superficial parafoveal RVD (%)	48.88 ± 1.57 (48.37–49.38)	48.82 ± 1.53 (48.33–49.31)	49.20 ± 1.50 (48.72–49.68)	0.668/**0.030**
	Superficial foveal RVD (%)	21.24 ± 4.99 (19.65–22.84)	20.97 ± 5.05 (19.35–22.58)	21.35 ± 5.00 (19.75–22.95)	0.405/0.744
	Deep overall RVD (%)	44.20 ± 1.41 (43.80–44.68)	43.87 ± 1.55 (43.52–44.51)	44.28 ± 1.52 (43.88–44.76)	**0.022**/0.568
	Deep parafoveal RVD (%)	50.90 ± 1.71 (50.42–51.59)	50.54 ± 1.75 (50.01–51.32)	51.03 ± 1.68 (50.58–51.58)	**0.031**/0.517
	Deep foveal RVD (%)	17.37 ± 4.59 (15.91–19.00)	17.19 ± 4.21 (16.12–18.59)	17.30 ± 4.30 (15.92–18.92)	0.619/0.865
Control (N = 18)	Superficial overall RVD(%)	41.66 ± 1.19 (41.07–42.25)	41.78 ± 1.27 (41.15–42.42)	42.19 ± 1.34 (41.53–42.86)	0.399/**0.008**
	Superficial parafoveal RVD (%)	47.04 ± 1.32 (46.38–47.70)	46.19 ± 1.77 (45.31–47.07)	47.56 ± 1.44 (46.84–48.27)	0.129/**0.015**
	Superficial foveal RVD (%)	20.14 ± 3.87 (18.21–22.06)	20.72 ± 4.17 (18.64–22.79)	20.73 ± 4.22 (18.64–22.83)	**0.036/0.036**
	Deep overall RVD (%)	42.19 ± 1.63 (41.38–43.00)	42.67 ± 2.01 (41.67–43.67)	43.08 ± 1.73 (42.22–43.95)	0.054/**0.002**
	Deep parafoveal RVD (%)	48.84 ± 1.59 (48.05–49.63)	49.38 ± 2.06 (48.36–50.41)	49.96 ± 1.81 (49.06–50.86)	0.072/**0.003**
	Deep foveal RVD (%)	15.56 ± 3.72 (13.71–17.41)	15.80 ± 3.71 (13.96–17.65)	15.57 ± 4.35 (13.41–17.74)	0.125/0.977

*RVD* retinal vessel density, *CI* confidence interval, *post–pre* comparisons by paired *t* tests for post-exercise vs. pre-exercise, rest-pre: comparisons by paired *t* tests for post-rest vs. pre-exercise.

Bold values indicate statistical significance: *p* < 0.05.

**Figure 3:**
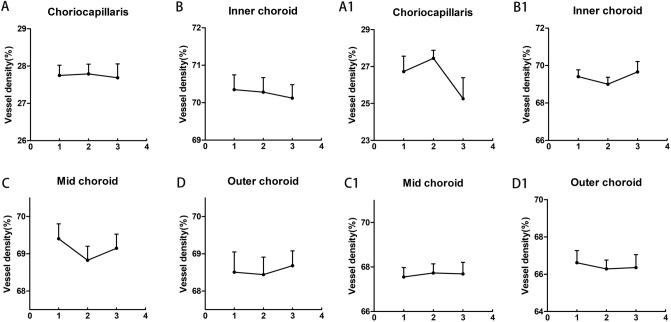
Changes in choroidal vessel densities (VD%) of myopic and emmetropic eyes in the choriocapillaris (**A**, **A1**), inner choroid (**B**, **B1**), mid-choroid (**C**, **C1**), and outer choroid (**D**, **D1**) across measurements obtained before exercise, immediately after exercise, and after the 30-min rest period. The error bars represent the standard error of the mean.

There were no significant CVD differences in slabs of the choriocapillaris, inner choroid, mid-choroid, and outer choroid during the study in both myopic and emmetropic eyes (Fig. 3A–D, A1–D1). However, the CVD in slabs of the mid-choroid tended to decrease after the exercise in myopic eyes, changing from 69.40 ± 2.47 to 68.83 ± 2.31% (*p* = 0.07, Fig. 3C).

### Correlations among SS-OCT, SS-OCTA, and hemodynamic parameters

**Figure 4:**
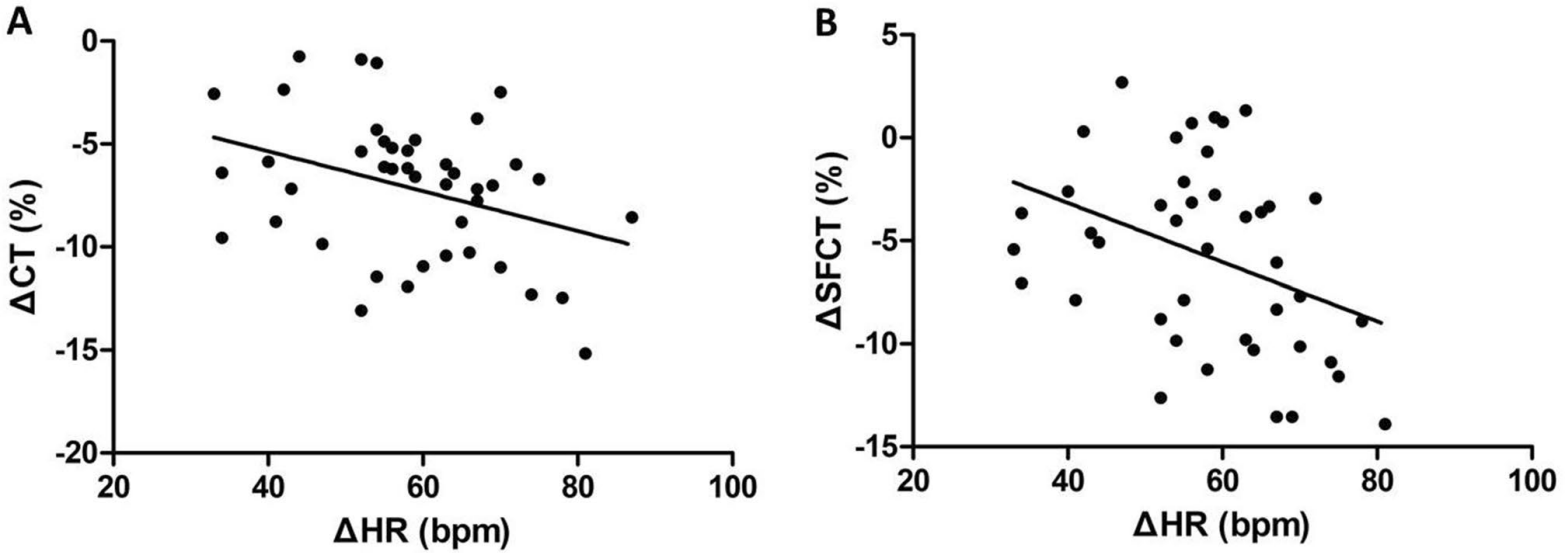
Scatter plot of ΔCT% against ΔHR (**A**) and scatter plot of ΔSFCT% against ΔHR (**B**) of myopic eyes. *CT* mean choroidal thickness; *SFCT* subfoveal choroidal thickness; *HR* heart rate.

Changes in HR were negatively correlated with changes in CT% (Pearson’s correlation r =  − 0.327, *p* = 0.04, Fig. 4A) and changes in SFCT% (Pearson’s correlation r =  − 0.345, *p* = 0.03, Fig. 4B) in myopic eyes. This correlation was not observed in emmetropic eyes. There were no significant correlations between ΔHR and other SS-OCTA parameters in both myopic and emmetropic eyes. No significant correlations were detected among ΔSBP, ΔCT%, and other SS-OCTA parameters.

## Discussion

In this study, we evaluated the effects of physical exercise on the CT and VD in the eyes of myopic and emmetropic children 9–13 years old, and we evaluated correlations among systemic dynamic parameters and the ocular changes. First, we observed that CT was significantly decreased after exercise in both myopic and emmetropic eyes, and this change continued to be significant after a 30-min rest period following the exercise. This finding differs from results reported for adults in which the CT was stable or even increased after exercise^11,12^. These inconsistencies may stem from the influence of the subjects’ health status, age, and exercise intensity. Alwassia et al.^11^ recruited patients who underwent cardiac stress testing, while Kinoshita et al.^12^ used a mild exercise protocol that may have had a limited effect on the choroid. We found that the HR was more than 66% of the age-dependent maximal HR immediately after exercise, indicating that the exercise intensity in this study was moderate or even more than moderate^16^. Our results showed a significant negative correlation of ΔHR with both ΔCT% and ΔSFCT% after exercise in myopic eyes, suggesting that the CT changes of myopic eyes may have been affected by exercise intensity. However, this correlation was not observed in the emmetropic eyes. In addition, the changes in CT% in myopic eyes were significantly larger than that in emmetropic eyes after completing the same exercise protocol. These different responses to exercise in myopic and emmetropic eyes may indicate differences in structure or function between these eyes.

The contractility of vascular and nonvascular smooth muscles and the elasticity of elastic fibers differ between children and adults, and aging can lead to arterial stiffening^17^. The choroid mainly consists of blood vessels and stroma, which contain nonvascular smooth muscle cells and elastic fibers^18^. In our study, we found that CT was decreased after exercise while CVD was relatively stable in both myopic and emmetropic eyes. The contraction of vascular and nonvascular smooth muscle cells induced by sympathetic excitation during exercise may be one of the reasons for the reduced CT^19^. In addition, dehydration induced by sweating may cause water in the matrix to enter blood vessels, thus compensating for the decrease in blood volume^20^. Considering all of these factors, we propose that 20 min of moderate physical exercise by children may lead to a significant decrease in CT that lasts for at least 30 min, while the water in matrix entering vessels maintained the CVD relatively stable. If the exercise time prolonged, whether CVD will continue to be stable with sweat loss is unknown. Previous studies have found that decreased CVD in myopic animal models is harmful to the retina and sclera, and that decreases in CT are related to the degree of myopia^21,22^. Therefore, whether or not a cumulative effect of exercise-induced choroidal thinning and decreased VD exists and whether or not this effect is harmful to the retina and sclera in children are questions requiring further study.

Our second important finding was that immediately after exercise, RVD was significantly decreased in the deep retinal layer in myopic eyes. This result was consistent with those presented in studies performed in adults in which macular perfusion decreased after exercise^10^. Ikemura et al.^20^ found that the blood flow in the internal carotid artery was significantly lower after exercise. They proposed that this phenomenon represented a reduction in the conductance index resulting from increases in blood viscosity, sympathetic activity, and the mean arterial partial pressure of CO_2_ (PaCO_2_) levels. In addition, the redistribution of blood to muscles and skin during exercise may also be involved in reducing the blood flow in the internal carotid artery, which may then influence fundus VD. Although autoregulatory mechanisms in the retina tend to maintain a constant blood flow over a wide range of perfusion pressures, blood flow can decrease beyond the compensation range^23,24^. Previous studies in adults showed that macular perfusion decreases in the superficial retinal layer after exercise, and that VD remains stable in the deep layer^6,20^. This discrepancy between the variation trend of RVD in superficial and deep layers after exercise may be attributable to differences in the elasticity and hardness of the fundus vessels between children and adults, which may lead to differences in autoregulatory ability^17^. As for the difference of RVD in deep layer between myopic and emmetropic eyes, it may also come from the different autoregulation capacity. Better vascular elasticity in emmetropic eyes results in faster RVD recovery after exercise before we can detect the fluctuation. Further animal studies may be needed to determine whether there is a difference in elasticity between retinal vessels in myopic and emmetropic eyes.

Researchers investigating functional hyperemia in the brain have suggested that arterioles are the primary sites of blood flow regulation^25^. In our study, when changes in BP were beyond the autoregulatory capacity of the retina, the RVD remained stable in the superficial layer while it decreased in the deep layer in myopic eyes. Based on this phenomenon, we infer that the arteries in the superficial layer, but not the deep layer, which consists of capillaries, tended to maintain a constant blood flow. Interestingly, the parafoveal RVD in the superficial layer in myopic eyes and RVD of all regions and layers in emmetropic eyes after the 30-min rest period were higher than the pre-exercise values. This effect may be induced by the recovery of PaCO_2_ due to breathing stabilization and discontinued hyperventilation after rest. The duration of this increase in RVD and whether or not additional benefits exist require further evaluation.

Our third important finding was that the IOP did not significantly change during the exercise and after the rest period, which is contrary to results presented for adults in previous studies showing a reduction in IOP after physical exercise^26,27^. However, the extent of IOP reduction has been found to vary among different types of exercises. The IOP-lowering effect of exercise seems to dissipate quickly, and this effect was very modest in most previous studies^28^. In our study, the children used a low-impact physical activity, cycling, for the exercise test, which did not induce a detectable reduction in IOP. We measured IOP after SS-OCT and SS-OCTA were performed, and the small delay may have weakened the exercise-induced IOP-lowering effect. Further, most children were nervous during the IOP measurement, and the anxiety may have elevated the IOP. In addition, the IOP could have been elevated if children held their breath during the cycling exercise^29^. All of the above factors may affect the evaluation of exercise induced IOP-lowing effect.

Some limitations to our study exist. First, we did not find an exercise protocol specifically designed for children; therefore, we relied on protocols used for adults in previous studies and basic guidelines for exercise tests^6,20,30^. The final exercise protocol was selected based on the performance of children in a preliminary study. Because we have recorded detailed data from this exercise protocol in children, future studies can make adjustments based on our data to meet their own needs. Second, we did not monitor systemic dynamic parameters in real time during exercise, and we evaluated exercise intensity based on the HR measured immediately after exercise. This protocol could have led us to underestimate exercise intensity. However, during the entire exercise process, a specialized researcher monitored the children and guided them to ensure that they maintained a constant pedaling speed. In addition, because SS-OCTA cannot detect blood flow velocity, a more accurate assessment of the changes in fundus blood flow was not possible.

In conclusion, moderate physical exercise for 20 min by children 9–13 years old significantly decreased the CT for at least 30 min after the exercise period and increased the fundus VD after rest. In addition, there was a significant difference of exercise induced CT thinning and RVD fluctuation between myopic and emmetropic eyes. Based on these results, we conclude that there are differences between children and adults, myopic and emmetropic eyes in the effects of exercise on fundus VD and CT. Further studies should clarify the mechanisms and effects underlying these differences.

## Methods

### Subjects and ophthalmic and systemic examinations

Forty myopic children (mean age: 11.70 ± 1.04 years; 23 males, 17 females) and eighteen emmetropic children (mean age: 11.06 ± 1.35 years; 12males, 6 females) aged 9–13 years were recruited from the Eye Hospital of Wenzhou Medical University for this study. The study was performed in accordance with the tenets of Declaration of Helsinki and was approved by the Ethics Committee of Wenzhou Medical University. Written informed consent was obtained from all the children and their parents after an explanation of the study content and methods.

Each subject underwent a comprehensive ocular examination, including slit-lamp examination, subjective refraction, AL measurement using a Lenstar LS900 (Haag-Streit AG, Koeniz, Switzerland), and intraocular pressure (IOP) measurement with a computerized tonometer (CT-1, Topcon Corporation, Tokyo, Japan). All examinations were performed in the above order. The inclusion criteria were best-corrected monocular visual acuity of at least 0.1 log (minimum angle of resolution), refractive error <  + 0.50 D and >  − 5.00 D, and IOP < 21 mmHg. After the ocular examination and a 10-min rest period, HR, SBP, and diastolic BP (DBP) were measured via an automated BP cuff before exercise.

**Figure 5:**
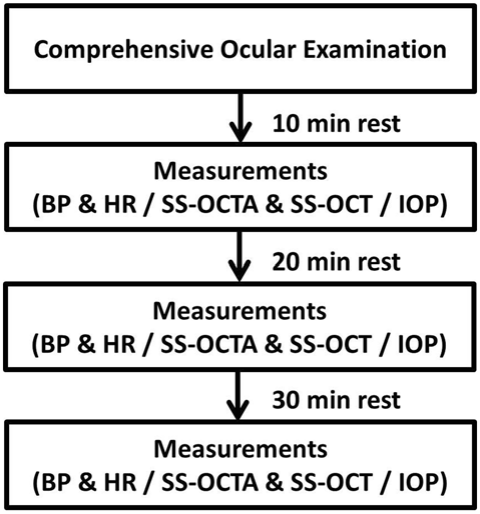
Flow diagram of all subjects through the study. *BP* blood pressure; *HR* heart rate; *SS-OCTA* swept source optical coherence tomography angiography; *SS-OCT* swept source optical coherence tomography; *IOP* intraocular pressure.

The physical exercise consisted of 20 min of riding a stationary bicycle (Merach Corporation, Hangzhou, China), and was performed under the supervision of a specialized researcher. The exercise was discontinued in the event of intense discomfort. Considering that children cannot bear heavy exercise loads, the protocol was adapted from previous studies of adult cycling exercise protocols^6^ and American Heart Association guidelines^15,31^. The exercise began with a warm-up period of 10 min during which the resistance was set at level 1, which was equivalent to 0 kg of resistance. During that period, the children were encouraged to maintain a speed of 20 km/h. In the second 10-min period, the resistance level was increased to level 2, which was equivalent to 0.5 kg of resistance, and the participants were encouraged to maintain a pedaling speed above 25 km/h. Handrail support was not encouraged during the cycling exercise because it may attenuate the physiological responses^32^. Given that heavy breathing after exercise negatively affects the quality of ocular imaging, we measured the SBP, DBP, and HR immediately within 1 min after the exercise was completed. We then obtained the SS-OCT and SS-OCTA images. The same measurements were repeated after a 30-min rest period (Fig. 5).

### SS-OCT and SS-OCTA measurements

The subjects were imaged using a DRI-Triton SS-OCT (Topcon Corporation, Tokyo, Japan) with a central wavelength of 1,050 nm, a scanning speed of 100,000 A-scans per second, and axial and transverse resolutions of 8 and 20 μm, respectively, in tissue. All SS-OCT and SS-OCTA images were acquired in follow-up mode and repeated three times by a well-trained examiner before and immediately after exercise and after the 30-min rest period.

Macular CT was obtained with the automatic built-in software of the SS-OCT device. The SS-OCT scan was a 6 × 6-mm radial scan centered on the fovea, with a scan resolution of 1024 × 12 pixels. Thickness maps were created according to the Early Treatment Diabetic Retinopathy Study (ETDRS) grid with nine independent subfields. The 1-mm diameter central region was centered on the fovea. The parafoveal regions (1–3 mm diameter, centered on the fovea) included the inner nasal, inner superior, inner temporal, and inner inferior regions. The perifoveal region (3–6 mm diameter, centered on the fovea) included the outer nasal, outer superior, outer temporal, and outer inferior regions. The SFCT was measured with a 9-mm line scan. Because the automatic techniques to quantify CT may be influenced by variability in anatomical features, manual correction was conducted by two experienced investigators when necessary^33,34^.

The SS-OCTA scan area was centered on the fovea and had a 3 × 3-mm field of view. Only images with a signal strength index above 55 were accepted. Any image with a significant motion artifact or a double-vessel pattern was excluded. For each subject, the right eye was considered first. If the pre-exercise SS-OCTA images of the right eye were of poor quality, then the left eye was selected instead. Only three left eyes were selected in this manner.

VD was defined as the percentage area occupied by perfused blood vessels in the scanned region. RVD measurements were performed in five subfields using the two innermost circles of an ETDRS grid overlay. The reproducibility of this method was assessed in a previous study and considered acceptable^35^. Default automatic segmentation distinguished the superficial retinal layers from 2.6 µm below the ILM to 15.6 µm below the IPL, while the offset for the deep retinal layers extended from 15.6 to 70.2 µm below the IPL.

**Figure 6:**
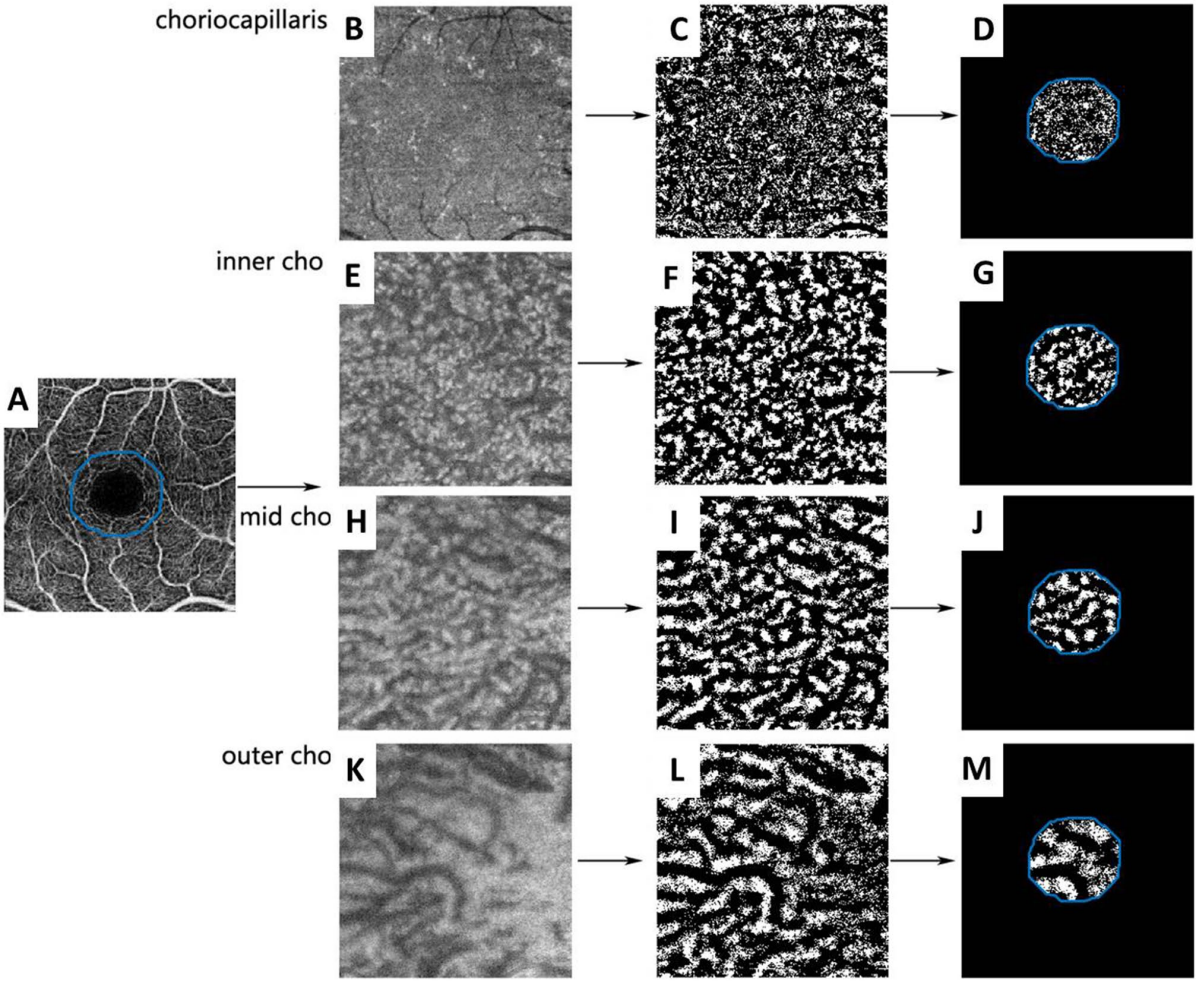
Representative exported and processed images. SS-OCTA and en face images were exported from the retinal superficial layer (**A**), choriocapillaris (**B**), inner choroid (inner cho, **E**), mid-choroid (mid cho, **H**), and outer choroid (outer cho, **K**). These images were first aligned to the retinal superficial layer and then binarized in ImageJ (**C**, **F**, **I**, **L**). An area of approximately 1/3 of each image, centered on the fovea, was selected to calculate choroidal vessel density (**D**, **G**, **J**, **M**). *SS-OCTA* swept-source optical coherence tomography.

The CVDs of the choriocapillaris, inner choroid, mid-choroid, and outer choroid were calculated from binarized en face SS-OCTA images as described in a previous study, with some modifications^36^. First, we exported the retinal superficial slab and en face images of the default choriocapillaris slab (0–10.4 μm). We then measured the SFCT based on each image and exported the en face images of the inner, mid-, and outer choroid at one-quarter, one-half, and three-quarters of the SFCT. Second, the three superficial retinal slabs of each subject were aligned, and an area centered on the fovea of approximately 1/3 of each image was selected. Third, the choriocapillaris slab and inner, mid-, and outer choroid slabs were binarized and analyzed with MATLAB. The choroidal VD of each slab was calculated as the percentage area occupied by the choroidal vessels in the selected area (Fig. 6).

### Statistical analysis

The CT, SFCT, and HR values immediately after exercise and after 30 min of rest following the exercise were normalized by subtracting the baseline (pre-exercise) values to obtain ΔCT, ΔSFCT, and ΔHR. ΔCT represented the mean CT of all selected areas. Because the ΔCT and ΔSFCT may be influenced by the baseline CT, we calculated the changing ratios of these parameters and obtained ΔCT% and ΔSFCT%. These two parameters were analyzed with independent t-tests. The MAP was determined by the following equation: MAP = [SBP + (2 × DBP)]/3^6^. All parameters were measured three times, and the mean values were calculated. Paired t-tests were used to analyze differences in RVD, CVD, ΔCT, ΔSFCT, ΔSBP, ΔDBP, and ΔHR between pre-exercise values and the post-exercise and post-rest values. Independent t-tests were used to analyze differences of all the measurement parameters and Chi-square test was used to analyze the difference of sex between myopic and emmetropic groups. The degrees of correlation between pairs of variables were expressed as Pearson’s correlation coefficients. We performed all statistical analyses with IBM SPSS Statistics 22 for Windows (IBM Corporation, Somers, NY, USA). Unless otherwise stated, data were presented as the mean ± standard deviation, after graphical confirmation that they met the normality assumption. For all findings, the selected level of statistical significance was a two-tailed *p* < 0.05.
